# Haemophilus parainfluenzae Infection Presenting as Prosthetic Joint Infection of the Shoulder, Vertebral Osteomyelitis, and Iliopsoas Abscess During Tocilizumab Therapy: A Case Report

**DOI:** 10.7759/cureus.89858

**Published:** 2025-08-12

**Authors:** Minoru Sakakiyama, Koji Hayashi, Rikuto Yoshimizu, Maho Hayashi, Katsunori Mizuno

**Affiliations:** 1 Department of Internal Medicine, Fukui General Hospital, Fukui, JPN; 2 Department of Endocrinology, University of Fukui, Fukui, JPN; 3 Department of Rehabilitation Medicine, Fukui General Hospital, Fukui, JPN; 4 Department of Orthopedics, Fukui General Hospital, Fukui, JPN

**Keywords:** haemophilus parainfluenzae, iliopsoas abscess, prosthetic joint infection (pji), shoulder joint, tocilizumab, vertebral-osteomyelitis

## Abstract

We describe the first case of prosthetic joint infection of the shoulder (PJIS), vertebral osteomyelitis, and iliopsoas abscess (IPA) caused by *Haemophilus parainfluenzae* (HPI). The patient was a 76-year-old male with a history of rheumatoid arthritis (RA) treated with tocilizumab (TCZ) and multiple prior surgeries for left prosthetic shoulder arthroplasty due to left degenerative shoulder osteoarthritis. He was admitted for shoulder and lower back pain. Imaging studies revealed dislocation of the left prosthetic shoulder joint and fluid accumulation suggestive of a dorsal hematoma, as well as a compression fracture at the T11 vertebra. Conservative treatment failed to alleviate the shoulder pain, and a humeral head replacement was subsequently performed. Intraoperatively, a purulent collection was observed in the area initially presumed to be a hematoma. On day 10 after admission, lumbar MRI revealed vertebral osteomyelitis at the L1/2 level and a left-sided IPA. The same day, surgical drainage via iliac fossa incision was performed, and a drain was placed. HPI was isolated from both the shoulder joint and lumbar specimens. The patient underwent two joint aspirations for recurrent shoulder swelling and received a combination of antibiotics, which were later de-escalated to levofloxacin and trimethoprim/sulfamethoxazole, and finally switched to oral amoxicillin-clavulanate before discharge. This case underscores the critical need for heightened vigilance and close monitoring for PJI, particularly during immunosuppressive therapy with agents such as TCZ and other biologics. Further accumulation of cases is needed to clarify the relationship between TCZ and HPI infections.

## Introduction

Tocilizumab (TCZ) is a monoclonal antibody that targets the interleukin-6 (IL-6) receptor and has become an effective treatment option for rheumatoid arthritis (RA) [1]. It was first approved in Japan for moderate to severe RA in 2005, followed by approval in Europe in 2009 and in the USA in 2010 [1]. Besides RA, TCZ is used to treat Crohn's disease, asthma, Castleman disease, and as an adjunct therapy for multiple myeloma, certain lymphomas, and some solid-organ cancers [2]. It is also employed palliatively for oral squamous cell carcinomas, multidrug-resistant anemia, and to manage cytokine storms in critically ill COVID-19 patients [2]. TCZ can suppress humoral immunity through IL-6 inhibition, leading to an increased risk of infections, particularly in the respiratory and urinary tracts [1]. Additionally, neutropenia is a common side effect associated with TCZ therapy [1]. Through these mechanisms, TCZ is associated with the development of infectious complications, including not only typical bacterial infections but also fungal infections such as candidiasis, aspergillosis, coccidioidomycosis, and *Pneumocystis jirovecii* pneumonia, which may form disseminated lesions [1]. Therefore, patients should be closely monitored for the development of infections during TCZ treatment.

*Haemophilus parainfluenzae* (HPI) is a pleomorphic Gram-negative coccobacillus with fastidious growth requirements, which require enriched media, usually containing blood (e.g., chocolate agar) [3]. It can be differentiated from other *Haemophilus spp.* by the requirement for V factor (i.e., NAD, nicotinamide adenine dinucleotide) for growth [3]. HPI is a common member of the human oropharyngeal and genitourinary microbiota, increasingly recognized as an opportunistic pathogen [4]. It can cause a variety of invasive, chronic, or recurrent diseases, including respiratory tract infections, meningitis, endocarditis and pericarditis, bone and joint infections, and arthritis [4]. Recent studies have also linked it to genitourinary and sexually transmitted infections, with high genital carriage and antibiotic resistance observed in pregnant women [4]. HPI is more frequently found than HPI in urethral exudates from men with urethritis, and is a common cause of urinary tract infections in boys with urinary tract abnormalities [4]. Urethritis caused by HPI has also been described in men who have sex with men, suggesting a role in sexually transmitted diseases [4].

HPI accounts for approximately 1-3% of cases of infective endocarditis [5]; however, reports of HPI-associated osteoarticular infections remain limited. To our knowledge, there have been no reports of concomitant pyogenic shoulder arthritis and iliopsoas abscess caused by HPI. We present a unique case of prosthetic joint infection of the shoulder (PJIS), vertebral osteomyelitis, and iliopsoas abscess (IPA) due to HPI in a patient receiving TCZ for RA.

## Case presentation

A 76-year-old male presented to our orthopedic department with complaints of left shoulder pain and low back pain. He had a 22-year history of RA, which had been treated with etanercept starting 17 years earlier and had been switched to TCZ the following year and continued thereafter. Twelve years prior, he had undergone total shoulder arthroplasty (TSA) for left degenerative shoulder arthritis. Due to implant dislocation associated with RA-related bone fragility, he had undergone revision reverse shoulder arthroplasty (RSA) twice: seven and six years ago.

**Figure 1:**
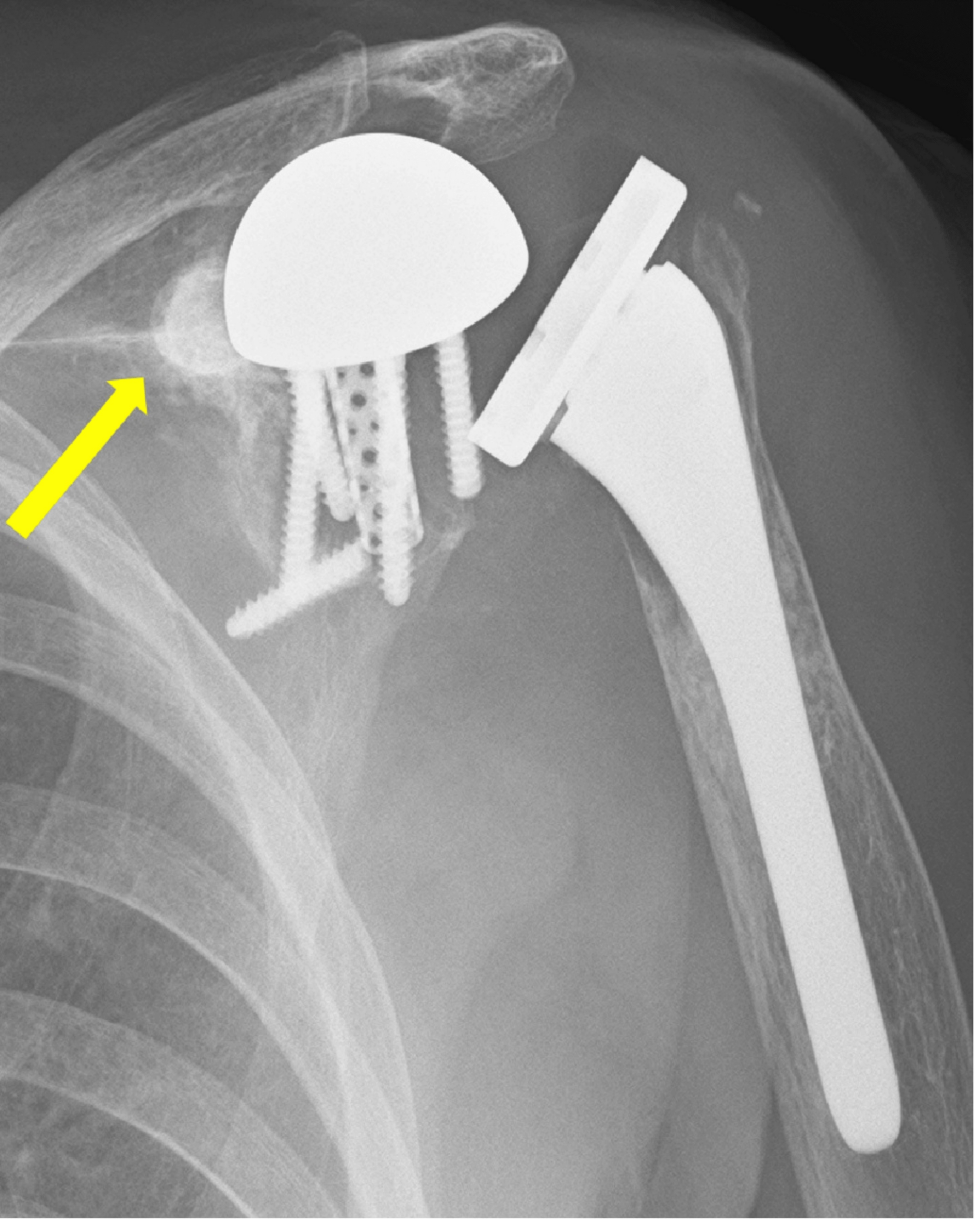
Shoulder X-ray findings Shoulder X-ray revealed dislocation of the left prosthetic joint of shoulder with fluid accumulation suggestive of a dorsal hematoma (arrow)

**Figure 2:**
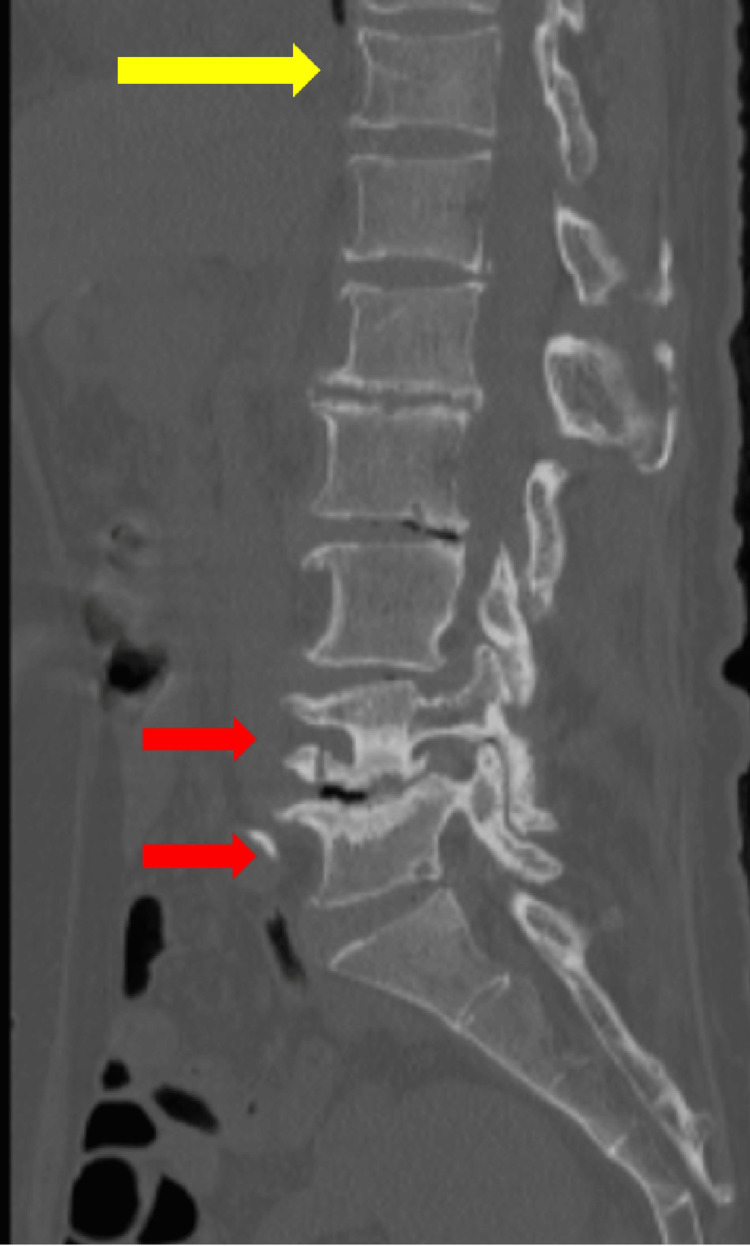
Lumbar CT findings Lumbar CT without contrast agent demonstrated a compression fracture at the T11 vertebra (yellow arrow) as well as L4 and L5 vertebrae (red arrows) CT: computed tomography

The shoulder X-ray revealed dislocation of the left prosthetic joint of the shoulder with fluid accumulation suggestive of a dorsal hematoma (Figure 1), and CT of the lumbar spine without contrast agent demonstrated a compression fracture at the T11 vertebra (Figure 2). Blood tests revealed elevated levels of white blood cells, C-reactive protein (CRP), and blood urea nitrogen, as well as decreased hemoglobin, platelets, and potassium (Table 1). He was admitted for further evaluation and treatment. Conservative management did not improve his shoulder pain, and on day six after admission, revision surgery was attempted. Intraoperatively, a significant amount of purulent fluid was observed at the site initially presumed to be a hematoma, indicating periprosthetic joint infection (PJI). All implants were removed, the site was thoroughly irrigated with iodine, and humeral head replacement was performed.

**Table 1:** Blood test results on admission

Parameter	Result	Reference range
Red blood cell (RBC)	362 × 10⁴/μL	(435-555 × 10⁴)
White blood cell (WBC)	14,300/μL	(3,300-8,600)
Hemoglobin	11.8 g/dL	(13.7-16.8)
Platelet (PLT)	10.1 × 10⁴/μL	(15.8-34.8 × 10⁴)
WBC differential, %		
Neutrophils	0.843	(40.0-70.0)
Lymphocytes	0.042	(25.0-45.0)
Monocytes	0.109	(2.0-7.0)
Eosinophils	0.004	(1.0-6.0)
Basophils	0.1	(0.0-1.0)
Total protein (TP)	5.5 g/dL	(6.6-8.1)
Blood urea nitrogen (BUN)	24.3 mg/dL	(8.0-20.0)
Creatinine (Cre)	0.63 mg/dL	(0.65-1.07)
Sodium (Na)	142 mmol/L	(138.0-145.0)
Potassium (K)	3.4 mmol/L	(3.6-4.8)
Chloride (Cl)	104 mmol/L	(101.0-108.0)
Estimated glomerular filtration rate (eGFR)	68.6 mL/min/1.73 m²	(60-89)
C-reactive protein (CRP)	5.06 mg/dL	(0.0-0.14)

**Figure 3:**
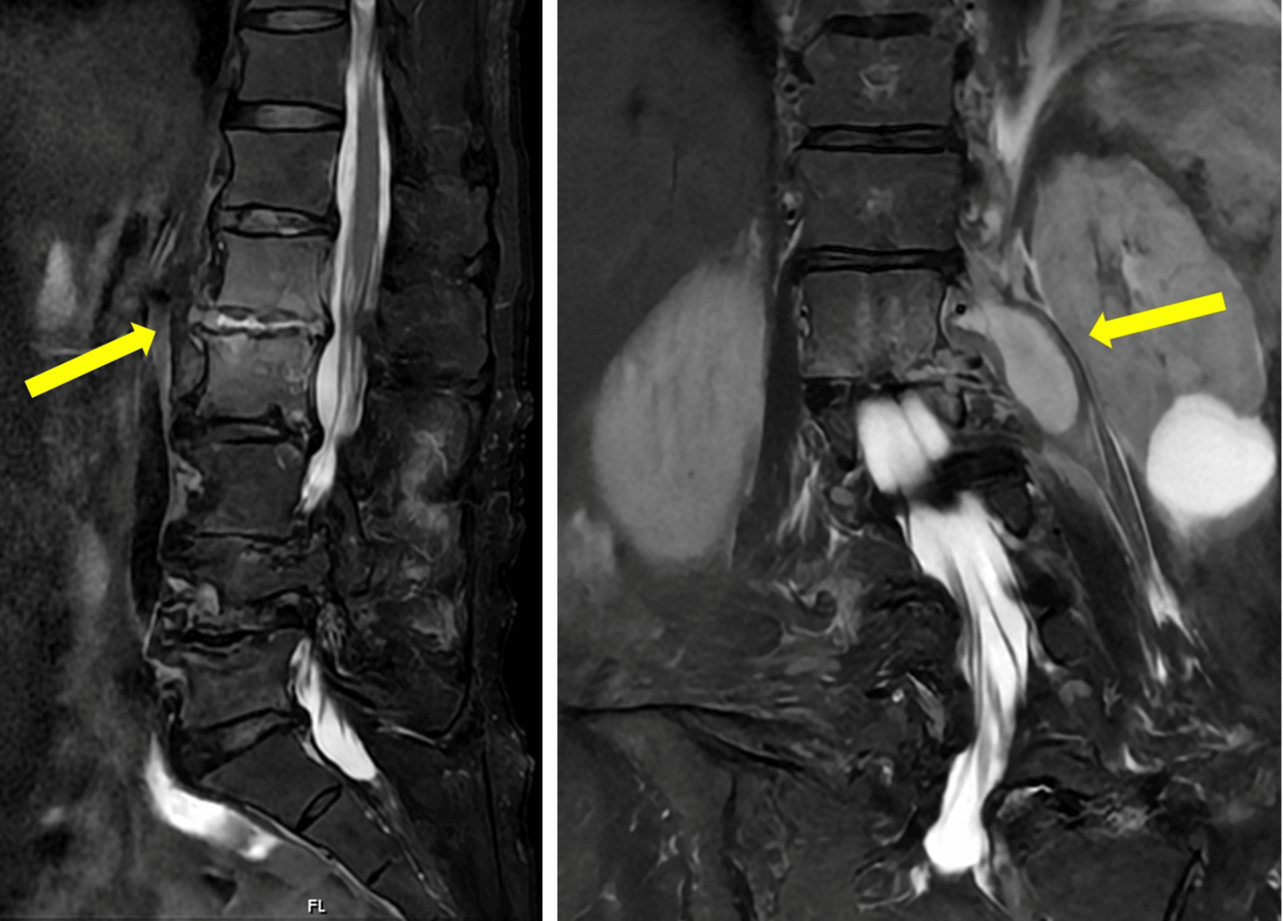
Lumbar MRI findings Lumbar MRI revealed hyperintensities at the L1/2 level (A) and a left-sided iliopsoas (B), indicative of vertebral osteomyelitis and iliopsoas abscess MRI: magnetic resonance imaging

On day 10, lumbar MRI revealed hyperintensities at the L1 and 2 vertebrae and a left-sided iliopsoas (Figure 3). Immediately, the patient underwent irrigation of the intervertebral disc and percutaneous drainage of the iliopsoas abscess. HPI was isolated from both the shoulder joint and the iliopsoas abscess specimens. Intervertebral disc cultures were negative, and blood cultures were not obtained. Based on these findings, he was diagnosed with PJIS, vertebral osteomyelitis, and an iliopsoas abscess caused by HPI.

TCZ and methotrexate were discontinued, and antimicrobial therapy was initiated with levofloxacin (LVFX), clindamycin (CLDM), and rifampicin (RFP). After identification of the pathogen and the sensitivity test results, trimethoprim-sulfamethoxazole (TMP/SMX) was added (Table 2). The patient subsequently showed improvement in inflammatory markers (including CRP and leukocytosis) and pain. On day 27, antibiotic therapy was de-escalated to a combination of LVFX and TMP/SMX. Multiple joint aspirations were performed thereafter for recurrent swelling of the left shoulder, but inflammatory findings were minimal, and all aspirate cultures remained negative. On postoperative day 78, antibiotics were temporarily discontinued due to suspected drug eruption. Oral amoxicillin-clavulanate (AMPC/CVA) was initiated on postoperative day 93, and the patient was discharged home in good condition on postoperative day 104 after a favorable course of rehabilitation.

**Table 2:** Antibiotic susceptibility profile

Antibiotic	Minimum inhibitory concentration	Sensitivity
Ampicillin	0.25	S
Ampicillin-sulbactam	<0.5	S
Cefotiam	1	S
Ceftriaxone	<0.12	S
Cefditoren pivoxil	<0.03	S
Cefepime	<0.25	S
Meropenem	0.25	S
Cefmetazole	<1	S
Clarithromycin	4	S
Ciprofloxacin	<0.12	S
Levofloxacin	<0.12	S
Trimethoprim-sulfamethoxazole	<0.25	S
Rifampicin	1	S

## Discussion

We reported the first case of HPI causing PJIS, vertebral osteomyelitis, and IPA. The patient used TCZ for the treatment of RA, but neutropenia was not observed. HPI was isolated in abscesses from the shoulder joint and the iliopsoas. Although a blood culture was not performed, the infection affecting other organs, such as PJIS, IPA, and vertebral osteomyelitis, suggested that the infection was likely disseminated via the bloodstream. The patient was treated with multiple antibiotics and was discharged with good outcomes.

We summarize the characteristics of infections during TCZ use. Based on the results of TCZ studies such as the TOWARD study, RADIATE trial, and STREAM trial, the incidence of serious infections was reported to be 5.7 cases per 100 patient-years [1,6,7]. In adults, respiratory infections were the most common type, followed by urinary tract infections and sepsis. Additionally, opportunistic infections such as candidiasis, aspergillosis, and pneumocystis pneumonia, as well as herpes zoster, hepatitis B virus reactivation, and tuberculosis reactivation have been reported [8]. Regarding the mechanisms underlying the increase in infections, it has been reported that host defense functions are impaired due to inhibition of the IL-6 pathway, that the impact on humoral immunity is more significant than on cellular immunity, and that there is a tendency toward recurrent infections (particularly upper respiratory tract infections).

To our knowledge, there have been only six reports of PJI caused by HPI [9-14]. Four of six cases involved compromised hosts or patients with a history of dental treatment. Five cases involved knee joints and one involved a hip joint. There have been no reports of cases involving shoulder PJI, vertebral osteomyelitis, and IPA caused by HPI, making this the first such report. Regarding PJIS risk factors, the overall PJIS rate is 2.4%, but RSA shows an infection risk up to 6.11 times higher than TSA due to increased implant surface area, and revision surgery has a higher infection risk than initial surgery, reaching up to 32% in some cases [15]. In PJIS, three main causative pathogens are recognized: *Cutibacterium acnes (C. acnes)* as the most common (38.9%), followed by *Staphylococcus epidermidis* (14.8%), and *Staphylococcus aureus* (14.5%) [16].

In our case, the likely pathophysiological route of infection was hematogenous spread, whereby bacteria originating from a distant site enter the bloodstream and seed the prosthetic joint, leading to infection. This mechanism is well recognized in late prosthetic joint infections occurring months to years after surgery [17,18]. TCZ, an IL-6 receptor antagonist, induces immunosuppression by inhibiting crucial immune pathways, thereby impairing host defense and facilitating the hematogenous dissemination of pathogens to the prosthesis [18]. Risk factors for PJI include prior revision surgeries and immunosuppressive therapy. Revision arthroplasties disrupt local tissue and vascular barriers, increasing vulnerability to bacterial colonization [19]. Concurrent immunosuppression, as in this patient receiving TCZ, further elevates the risk by diminishing systemic immune surveillance and bacterial clearance [19]. Together, these factors might contribute to the development and progression of the hematogenous PJI observed in this case.

HPI has been shown to exhibit antimicrobial resistance. Macrolides, tetracyclines, and some beta-lactam antibiotics have been reported to have limited efficacy against this bacterium [20]. Therefore, antimicrobial susceptibility testing is essential. Due to this drug resistance, previously reported treatments have involved administering quinolones, rifampicin, cephalosporins, and other drugs for periods ranging from six weeks to two years. In our case, drugs were selected based on drug susceptibility results. The reason for combining four types of antibiotics was that *C. acnes*, the most common causative organism in PJIS and capable of forming biofilms, was covered by the combination of clindamycin and rifampin [21], and additionally, the combination of levofloxacin and TMP/SMX was used due to their good penetration into abscesses [22,23]. Since *C. acnes*, an anaerobic bacterium, is difficult to isolate by culture [21], coverage was continued during the acute phase to account for the possibility of false-negative results. However, antibiotic selection could have been improved. For example, a narrower-spectrum antibiotic such as amoxicillin (AMPC) rather than AMPC/CVA may have been more appropriate.

This report has a limitation that needs to be acknowledged. Blood cultures, which are the gold standard for suspected disseminated infection, were not performed because the patient did not exhibit a high fever. Additionally, at our institution, blood cultures are only obtained if the patient’s temperature reaches 38.0 °C or higher (typically before antibiotic treatment), which was not the case here. If this information had been available, the presentation would have been more compelling.

## Conclusions

We described the first case of PJIS, IPA, and vertebral osteomyelitis via blood flow caused by HPI in the setting of immunodeficiency after artificial shoulder joint replacement. This report underscores the critical need for heightened vigilance and close monitoring for PJI, particularly during immunosuppressive therapy with agents such as TCZ and other biologics. Further accumulation of cases is needed to clarify the relationship between TCZ and HPI infections.
